# The Effectiveness of the Digital Environment and Perfectionism on Anxiety and Depression in the Light of the COVID-19 Pandemic in Northern Iraq

**DOI:** 10.3389/fpsyg.2022.804071

**Published:** 2022-05-17

**Authors:** Shahla Ali Ahmed, Yagmur Cerkez

**Affiliations:** Department of Psychological Counselling and Guidance, Ataturk Faculty of Education, Near East University, Nicosia, Cyprus

**Keywords:** digital environment, COVID-19, multidimensional perfectionism, anxiety, depression, educational staff, college student

## Abstract

This investigation is intended to explore the effect of the digital environment as well as perfectionism during the COVID-19 pandemic on anxiety and depression. The study used a mixed-methods design; a mixed research methodology was used regarding explanatory design by using a qualitative sub-sample from quantitative sample data. The researcher conducted the study on a sample of 980 students and non-students smartphone and internet users using both qualitative (self-reported) and quantitative (questionnaires) approach. Three different questionnaires were used: The Multidimensional Perfectionism Scale, Beck Depression (BD), and Beck Anxiety Inventory (BA). On the other hand, the researcher designed a self-reported interview for the qualitative part that included 5 major questions and 10 sub-questions. The gathered data were investigated using SPSS version 22 to analyze the collected data for this study, and simple descriptive statistics and coding were used. The results denote that the digital environment is significantly correlated with multidimensional perfectionism in a low positive manner for students. However, the correlation effects for non-students are significantly high as evidenced by a significant positive correlation. Another finding suggests that there is a positive significant association between perfectionism and being depressed and anxious.

## Introduction

The world has undergone an unprecedented ordeal last year as a result of the COVID-19 pandemic. It expanded rapidly across the globe, causing substantial worries, fears, and anxieties. There has been a considerable disruption in the lives of millions of people across the planet. Concern has also been expressed over the psycho-social and mental health impacts of the pandemic by the World Health Organization (WHO). Novel procedures like quarantine and self-isolation have disrupted normal routines, livelihoods, and activities, and it has been speculated that this could lead to a rise in depression, self-harm, loneliness, insomnia, suicidal behavior, anxiety, and harmful drug and alcohol use (World Health Organization, 2020). The latest Indian Psychiatric Society investigation showed a 20 percent rise in mental illnesses since the emergence of the coronavirus pandemic in India (Loiwal, 2020). The lives of people have changed completely due to social isolation and this had both psychological and technical consequences including effects on social and psychosocial well-being. The implementation of anti-epidemic measures, particularly the lockdown, has affected social contacts and daily activities and has also caused increased feelings of isolation, misuse of alcohol, drugs, and depression. On the other hand, the digital environment has continued to develop and has a huge influence on daily life. While the impact of the digital and techno environment during the COVID-19 pandemic on daily lives is clear, social networks and the mass media have also significantly raised fears. In a video message, the secretary-general of the United Nations (UN) highlighted psychological issues, such as isolation and depression as “being the greatest causes of unhappiness today” (Iancheva et al., 2020). Nine out of ten people have internet access at home (Antoniou, 2018), and wearable technologies, such as fitness trackers, are owned by 20% of households. Portability is one of the most essential aspects of a digital environment, as wearable technology is designed to be taken anywhere the person goes (Chayko, 2017). A major issue for modern society is smartphone dependence, as different undesirable results, including both social and psychiatric issues, may arise as a result (Demirci et al., 2015; EnezDarcin et al., 2015). The diverse applications of digital interventions and their potential effect on medical care, worldwide distribution of psychological therapies, and clinical services are considered (Abdullah et al., 2016). The term social media denotes the abundant internet-based networks that enable operators to communicate visually and orally with others (Carr and Hayes, 2015). At least 92% of adolescents engage in social media, as stated by the Pew Research Centre (2015). The 13–17 age group was described by Lenhart et al. (2015) as being particularly frequent users of social media, where 58% have access to a tablet device and 87% have access to a computer. Nearly three-quarters of teenagers between the ages of 15 and 17 use smartphones, while 68% of adolescents aged 13–14 use a smartphone (Pew Research Centre, 2015). Living in this world with such issues may be different for high-achievers (perfectionists). Perfectionism as a concept is complicated, because, in addition to its different functional effects, it is also multidimensional. Positive outcomes are linked to some of its facets; however, it is also associated with negative consequences (Macedo et al., 2014). Numerous studies have shown that perfectionism is a significant risk factor for depression, as well as anxiety (Blankstein and Dunkley, 2002). Therefore, during COVID-19, the recent growth of the digital environment, especially the internet and smartphones, has had significant psychological and social effects on the population. On the other hand, the unexpected health crisis that emerged last year due to the COVID-19 pandemic has played a significant role in changing the lives of people around the world, particularly in terms of their psychology. The globe is expected to face a significant mental health crisis in the aftermath of the COVID-19 epidemic (Galea et al., 2020). Even after the first wave of infection has passed, COVID-19-related stresses, such as self-quarantine, social isolation, job loss, and the danger of sickness, will linger. These elements are likely to have a profound effect on the human psyche, contributing to a secondary mental health epidemic (Fiorillo and Gorwood, 2020). The incidence of mental health sequels connected with the COVID-19 pandemic has been documented in numerous research conducted in a variety of countries (Brailovskaia et al., 2021). Some people are dealing with a significant psychological burden: they are feeling overwhelmed by their current condition, which leads to a negative emotional response marked by frustration, anxiety, and uncertainty. As a result, individuals are having substantial difficulties managing their daily lives and fulfilling their commitments under the current circumstances (Taylor et al., 2020). Previous research has shown that depressive symptoms have increased over the last decade, particularly among the younger population. Brailovskaia and Margraf (2020): worried of becoming ill and spreading the disease to their family (Huang et al., 2020), and burnout and a lack of well-being were also noticed (Busch et al., 2021). During the COVID-19 pandemic, recent studies anticipated that symptoms would worsen (Galea et al., 2020). Feelings of control loss, hopelessness, and helplessness describe people with increased levels of depressive symptoms (Lei et al., 2016). Pappa et al. (2020) found a significant prevalence of anxiety, depression, and sleeplessness during the COVID-19 pandemic in their systematic review and meta-analysis of 13 cross-sectional studies. However, people who are perfectionists and high-achievers usually present symptoms of perfectionism. Researchers have shown and highlighted the impact of this global problem on psychological health and well-being. One of the most exposed groups during any change includes students and educational staff. From this perspective, the fundamental objective of this study is to determine the effect of the digital environment and perfectionism on depression and anxiety among students and non-students in northern Iraq.

### Aim of the Study

The examination of perfectionism on psychological disorders (Anxiety and hopelessness) and the effectiveness of digital environment during coronavirus COVID-19 is the aim of this investigation.

The goal would, therefore, be to answer the following research questions:

(1) The effect of demographic information like (hours spend on internet and duration that use internet and smartphone, age, education level, and gender) will be investigated.
(2) Is there a substantial correlation between perfectionism, depression and anxiety, and the digital environment during the COVID-19 pandemic?

Research Hypothesis:

• H1 COVID-19 has a significant effect on digital environment (using smartphone and Internet);
• H2 COVID-19 has a significant effect on mental health;
• H3 COVID-19 has a significant effect on multidimensional perfectionism;
• H4 digital environment has a significant effect on mental health;
• H5 multidimensional perfectionism has a significant effect on mental health.

### Strengths of the Study

A large sample size and analyzing big qualitative data with 980 participants by using coding from thematic approach are one of the strengths of this study. The method design for this study is both qualitative and quantitative. Explanatory design consists of gathering statistical information using quantitative data and qualitative data to explain the variable and combine with statistics collected from quantitative and qualitative analysis (Creswell, 2011). With that, choosing the education sector as the part of the population that has been the most affected and most involved in this pandemic. When the entire world was shut down, they were doing the most work online, they try to deal with a new way of teaching learning-distance education.

## Methodology

### Research Design

Mixed research methodology will be used, sequential designs regarding explanatory design, and qualitative sub-sample from quantitative sample data (the Explanatory Design includes first gathering quantitative data and then gathering qualitative data to clarify or elaborate on the quantitative outcomes), as stated by Creswell (2011). Quantitative data from a structured survey questionnaire and qualitative data from a self-reported interview on participants in northern Iraq will be used.

#### Research Questions

The goal would therefore be to find answers to the following research questions:

Is there a significant correlation between perfectionism, depression and anxiety, and the digital environment during the COVID-19 pandemic?

#### Quantitative Research Question: For This Purpose Researcher Used Instrument (Questionnaire) to Measure the Variables

##### Research Questions

• Is there a location effect on digital education, perfectionism, and mental health?
• Is there a significant relationship between digital environments, perfectionism with anxiety, and depression?
• Is there a significant relation between perfectionism with anxiety and depression in the time of coronavirus?
• What is the level of and anxiety and depression among student Internet and smart phone user during COVID-19? Insights of student mental health.
• Does digital environment have a role to play between COVID-19 and mental health?
• Does multidimensional perfectionism have a role to play between COVID-19 and mental health?

The qualitative research questions were prepared by the researcher and five main questions were used after expert review.

Research Question One: What are the perceptions of internet and Smartphone users about the effectiveness of digital environment on anxiety and depression during COVID-19?

Research Question Two: What are the perceptions of internet and smartphone users about the digital environment during COVID-19? I feel this question should be removed also, because it is similar or identical with the question above.

Research Question Three: What are the perceptions of internet and smartphone users about the influence of perfectionism in the time of coronavirus?

Research questions four: How does perfectionism influence anxiety and depression during COVID-19?

Research questions five: What are the perceptions of internet and smartphone users about the relationship between digital environments, perfectionism, anxiety, and depression during COVID-19?

### Population and Sample

The study population includes student and non-students who have access to Internet and smartphones from Northern Iraq. Inclusion and exclusion criteria for the study are shown in Table 1. The participants could be from any culture, nationality and gender and were selected randomly. First, as the quantitative approach, we distributed 1,000 questionnaires among a random sample of students and non-students, Internet and smart-phone users, were 980 were completed. Secondly, the researcher collected 100 self-reported interviews in which 10 sub-questions for the 5 main research questions that were prepared by the researcher were answered to collect the qualitative data.

**TABLE 1:** Inclusion and exclusion criteria.

Inclusion	Exclusion
Adults ≥ 15 years of age	Any acute or chronic condition that would limit the ability of the patient to participate in the stud.
Smartphone user	Refusal to give informed consent
Minimize use internet for 1 h each day	Diagnosis of anxiety and depression disorder

### Demographic Profiling

As Table 2 shows the 497 students comprised of 264 male students and 233 female students, while the 483 non-student respondents comprised of 251 male non-students and 232 female non-students. A significantly higher number of students (*n* = 239) and non-students (*n* = 223) were in between the age group of 19–30 years. About 12.5% of the students and 10.8% of the non-students were at least 41 years old. Approximately 43.9% of the students and 42.9% of the non-students were graduates, while 18.9% of the students and 16.6% of the non-students were drop-outs. There is relatively high internet usage levels among students (7–9 h = 180, 10–12 h = 82, and 12 h and above = 32). There is also relatively high internet usage levels among non-students (7–9 h = 144, 10–12 h = 43, and 12 h and above = 14). A total of 167 students had COVID-19 and 330 students were COVID-19-free, while 178 non-students had COVID-19 and 305 students were COVID-19-free. One hundred sixty-six students lived at the city center, while 331 lived outside of the city. On the contrary, 182 non-students lived at the city center, while 301 non-students lived outside of the city.

**TABLE 2:** Demographic profiling.

		Students		Non-students	
Variable	Description	Frequency	Percentage	Frequency	Percentage
Gender	Male	264	53.1	251	52.0
	Female	233	46.9	232	48.0
	Total	497	100	483	100
Age	15–18	60	12.1	71	14.7
	19–30	239	48.1	223	46.2
	31–40	136	27.4	137	28.4
	41-above	62	12.5	52	10.8
	Total	497	100	483	100
Education level	High school	29	5.8	43	8.9
	B.Sc.	156	31.4	153	31.7
	Graduate student	218	43.9	207	42.9
	Drop out	94	18.9	80	16.6
	Total	497	100	483	100
Hours spends on	1–3 h	57	11.5	136	28.2
internet in 24 h	4–6 h	146	29.4	146	30.2
	7–9 h	180	36.2	144	29.8
	10–12 h	82	16.5	43	8.9
	12 h and above	32	6.4	14	2.9
	Total	497	100	483	100
Use of online	Yes	327	65.8	318	65.8
education	No	170	34.2	165	34.2
	Total	497	100	483	100
COVID-19 illness	Yes	167	33.6	178	36.9
	No	330	66.4	305	63.1
	Total	497	100	483	100
Residential	Centre of the city	166	33.4	182	37.7
location	Outside of the city	331	66.6	301	62.3
	Total	497	100	483	100

### Data Collection Tools

There are three variables in the study: one independent variable is the perfectionism, and the two dependent variables are anxiety and depression. The investigator gathered data for the above-mentioned changes by means of three pre-formatted surveys.

The investigator modified the Beck et al. (1988) scale for measuring the anxiety levels among the participants. The scale is a collection of self-disclosing measures. The instrument that was used for this study was a questionnaire that measured the level of anxiety. This consisted of an initial number of 21 items with 4 options included [Not at all (1) Mildly, but it didn’t bother much (2), moderately- it wasn’t pleasant at time (3) and Severely-it bothered me a lot (4)] listing the common symptoms of anxiety.

The researcher adapted the Beck et al. (1961) scale for measuring the depression levels among the participants. The scale is a set of self-reported scales. The instrument that was used for this study was a questionnaire that measured the level of depression. This consisted of an initial number of 21 items with 4 options (starting from 0 to 3) that measure characteristic attitudes and symptoms of depression.

On the other hand, the researcher adapted the Multidimensional Perfectionism Scale of Hewitt and Flett (1989) with varying degrees of perfectionism traits: self-oriented perfectionism, other-directed perfectionism, and socially directed perfectionism, with 35 items and five options: (1) Strongly disagree, (2) disagree, (3) neither agree nor disagree, (4) agree, and (5) strongly agree for each of the items. Some substances are reverse-scored, and the subscales are counted in a way that higher score indicates superior perfectionism.

The researcher used self-reported interviews for the quantitative part that contained 5 major questions and 10 sub-questions, which were accepted by psychological experts to use. The questions require respondents to provide descriptive and in-depth answers to reach the aim of the study to identify the effectiveness of the digital environment and perfectionism during COVID-19 on anxiety and depression.

### Data Collection Procedure and Ethics

Before the researcher collected the data, suitable permission was obtained. The researcher did not harm any participants or contributors during the investigation. To determine the effect of the digital environment and perfectionism during COVID-19 on anxiety and depression, an explanatory design was applied, which included gathering qualitative data; afterward, a quantitative stage was conducted for clarifying or follow up on the quantitative data in greater depth. In the initial quantitative stage of the investigation, questionnaires were used. The questionnaire was distributed to 1,000 students and non-students internet and smartphone users and a total of 980 forms were collected. The data were used to analyze the effectiveness of the digital environment and perfectionism with respect to depression and anxiety during COVID-19. The second phase is the qualitative phase, which was conducted to better understand the effectiveness of the digital environment and perfectionism on depression and anxiety during COVID-19. The self-reported interview was answered by 100 participants. The study did not harm or exploit any of the participants, either emotionally or physically. The respondents also consented to the study and had the right to leave the study at whatever time they wished. Subsequently, the investigator divided the questionnaire among participants starting with the demographic information section and followed by the other sections. The research did not include any sensitive information and the names of the contributors were excluded. The researcher ensures that all sensitive issues are not included in the questionnaire to achieve the objective of the research. After the quantitative part, the participants were given the freedom to discuss their feelings and views about the subject and were advised that they could take a rest or break at any time.

### Data Analysis

Quantitative gathered data were examined by means of descriptive statistics to determine the magnitude of effect of the variables. The data analysis measures that were implemented comprised the application of descriptive statistics, like mean and standard deviation (SD), the goal of which was to evaluate the magnitude of influence, and also the responsiveness of the variables, respectively (Neuman, 2013). Pearson correlation coefficient tests were used to test the proposed hypothesis. The data were scrutinized by means of Statistical Packages for Social Sciences (SPSS), version 22. The researcher analyzed the data using various statistical tests. The reliability of the data was determined using Cronbach’s Alpha and the validity using construct reliability and Forknell-Lacker. The heterotrait-monotrait ratio was used to check similarities between items in the variables. Structural Equation Modeling (SEM) was applied to define relationships between the variables with Path analysis being used for the purpose of identifying the direction of the relationships.

#### Structural Equation Modeling Model

The statistical approach of SEM is used to quantify and analyze the relationships between observable and latent variables. It explores linear causal links between variables, while accounting for measurement error, similar to but more powerful than regression analysis (Beran and Violato, 2010). The multivariate analysis technique’s SEM is commonly employed in the social sciences (González et al., 2008). Its applications span from simple connection analysis to complicated measurement equivalence analyses for first and higher order entities (Cheung, 2008). It provides a flexible framework for constructing and analyzing complicated interactions between various variables, allowing researchers to use empirical models to test the validity of theories. The capacity to manage measurement error, which is one of the most significant drawbacks of most studies, is maybe its most significant advantage. Although it has been utilized in variety of fields, it has, yet, to be widely adopted in medical research and epidemiology.

#### Path Analysis

Path analysis is a method for determining and assessing the impacts of a collection of factors acting on a defined outcome *via* numerous causal routes. It is a forerunner to and subset of structural equation modeling. A statistical technique for analyzing and testing links between a set of observed variables is path analysis. Path analysis allows for the simultaneous investigation of several direct and indirect interactions between variables (Valenzuela and Bachmann, 2017).

The qualitative data gathered for answering the research questions were examined by means of plain descriptive statistics and coding, and a thematic approach was applied to code and analyze the self-reported results using Nvivo software. The reported result indicates the views of internet and smartphone users about the effect of the digital environment on anxiety and depression during COVID-19, in line with the goal and objective of the research. Tables, graphs, and pie charts were used to show the results.

## Results

### Findings Related to Research Question 1

#### Location Effects on Digital Education, Perfectionism, and Mental Health

Studies have rarely considered the influence of location on aspects like digital education, perfectionism, and mental health, and the aim of this study is to fill this gap. It is vital to address questions and problems related to the effectiveness of the digital environment and perfectionism during the COVID-19 pandemic on mental health (anxiety and depression). Subsequently, an examination of these influences was carried out with regard to how location influences digital education, perfectionism, and mental health.

#### Location Effects on Digital Education

Studies have rarely considered the effects of location on sociological, cultural, and educational aspects. One question of the research was aimed at exploring whether location influenced the adoption of digital education among students and non-students in the Kurdistan region of Iraq. This denotes the study’s novelty and originality, and this was accomplished by the application of the independent *t*-test. The computed independent *t*-test results showed that location had no influence on the use of digital education by both students (*p*-value > 0.05) and non-students (*p*-value > 0.05).

Therefore, these findings imply that the adoption of digital education in the Kurdistan region of Iraq was primarily influenced by economic development, technological innovation, and structural factors like COVID-19 (Siron et al., 2020). This is a reflection of practical situations prevalent in the country because studies consider the adoption of digital education to be influenced by economic development (Szopiński, 2016) and technological innovation (Salinas et al., 2017).

#### Location Effects on Multidimensional Perfectionism

The study findings presented in Table 1 showed that the students’ multidimensional perfectionism was not influenced by their location (*p* > 0.05). Meanwhile, considerable differences between students and non-students were observed as the location significantly influenced multidimensional perfectionism among non-students (*p* > 0.05).

Attention was also given to determine whether location effects influenced multidimensional perfectionism. This study shows that multidimensional perfectionism varies significantly according to social structures, values, and norms. In addiction socially prescribed, self-oriented, and other-oriented perfectionism show differences according to the participant places of residence (Hewitt and Flett, 1989). This finding contradicts Hewitt and Flett (1989). This may imply that family values and individual differences had a huge effect on multidimensional perfectionism among students. Meanwhile, considerable differences between students and non-students were observed as location significantly influenced multidimensional perfectionism among non-students (*p* > 0.05). This aligns with the findings of Hewitt and Flett (1989) that self-oriented and other-oriented perfection are influenced by factors like cultural and religious orientation. This is certainly true in Kurdistan, which is composed of various ethnic and religious groups like Suni, Kurdish Bandini, Sorani, Yazidi, etc.

#### Location Effects on Mental Health

The mental health of both students and non-students was largely the same, irrespective of differences, due to geographical location. The independent *t*-tests revealed that there were insignificant differences in depression levels among students (*p* > 0.05). However, the students were significantly anxious about COVID-19, as the results of Table 3 shows that the students’ anxiety levels measured by the Beck Anxiety Inventory (BAI) were significantly different (*p* < 0.05).

**TABLE 3:** Location effects on mental health, digital education, and COVID-19.

Variables	Students			Non-students		
	*F*	Df	*p*-value	*F*	Df	*p*-value
BDI*	1.451	495	0.888	0.003	481	0.590
BAI*	2.511	495	0.003	0.004	481	0.233
Digital education	2.259	495	0.756	4.620	*481*	0.344
Perfectionism	2.216	495	0.446	4.620	481	0.089

*BDI, Beck depression Inventory and BAI, Beck Anxiety Inventory.

This result is valid not only to people in Kurdistan, but the world, in general, remains anxious about the development of effective solutions that will end COVID-19 (He et al., 2021).

Relatively similar observations were observed for non-students [Beck depression Inventory (BDI): *p*-value = 0.590]. This reveals that there were insignificant differences in depression levels among non-students. Different results were observed regarding the location effects of COVID-19 on non-student’s anxiety levels. In other words, the location effects of COVID-19 on non-student’s anxiety levels were insignificant (BAI: *p*-value = 0.233). This possibly implies that uncertainties surrounding COVID-19’s prevalence, as well as new variants, effects, and eradication caused students to be more anxious compared to non-students.

### Findings Related to Research Question 2

#### Correlation Effects Between Digital Environment, Perfectionism, and Mental Health

The Pearson correlation coefficient test was conducted to examine how digital education, perfectionism, and mental health are correlated. The results in Table 4 denote that digital environment is significantly correlated with multidimensional perfectionism in a low positive manner for students with a value of 0.453. However, the correlation effects for non-students are significantly high as evidenced by a significant positive correlation of 0.722.

**TABLE 4:** Correlation effects.

Participants		Students			Non-students		
Variables		DE	MP	MH	DE	MP	MH
DE	Pearson correlation	1			1		
	Sig. (two-tailed)	–			–		
	*N*	497			483		
MP	Pearson correlation	0.453**	1		0.722**	1	
	Sig. (two-tailed)	0.000	–		0.000	–	
	*N*	497	497		483	483	
MH	Pearson Correlation	0.358**	0.621**	1	–0.089*	0.005	1
	Sig. (two-tailed)	0.000	0.000		0.049	0.912	–
	*N*	497	497		483	483	483

**Correlation is significant at the 0.01 level (two-tailed).

This suggests that digital environment enhances more non-students’ awareness of socially prescribed and other-oriented perfectionism than students (Hewitt and Flett, 1989).

Improvements or the adoption of digital environment were positively connected to improvements in both students’ and non-student’s mental health; for student’s by 0.358. This is because the digital environment helps students to acquire more information about COVID-19, anxiety, or depression. This causes them to adopt effective measures to reduce them and, subsequently, improves their mental health. On the contrary, a significant negative correlation of –0.089 was observed between non-students’ digital environment and mental health. This suggests that digital environment’s improvements were being used for non-productive activities or purposes that adversely affected non-students’ mental health. For instance, non-students may devote a substantial amount of time off to social media, playing games, as well as other irrational activities, which causes them to be sleep-deprived and, thus, negatively affects their mental health.

Significant and non-significant positive correlations of 0.621 and 0.912 were observed between multidimensional perfectionism and mental health among students and non-students, respectively. This suggests that multidimensional perfectionism aspects, especially self-orientation of an individual’s issue, affect their well-being and happiness (Gnilka et al., 2012). As such, they can take appropriate steps to ensure that these circumstances do not undermine their mental health. Furthermore, studies have shown that multidimensional perfectionism aspects like socially prescribed and other-oriented perfectionism aid individuals in avoiding circumstances that adversely affect their mental health (Mathew et al., 2014; Mateen et al., 2020). Gnilka et al. (2012) established that multidimensional perfectionism helps people to copy with anxiety.

### Findings Related to Research Question 3

Is there a significant relation between perfectionism, anxiety, and depression during the coronavirus pandemic?

The findings of the study suggest that there is a positive, but significant correlation between perfectionism, depression, and anxiety (*P* < 0.01, *r* = 0.346^**^ and *P* < 0.05, *r* = –0.270). This means that during COVID-19, any change in perfectionism will cause significant changes in depressions and anxiety, as shown in Table 5.

**TABLE 5:** Correlation analysis.

Variables		Perfectionism	Anxiety	Depression
Perfectionism	Pearson correlation	1	0.270*	0.346**
	Sig. (two-tailed)		0.02	0.00
	*N*		980	980
Anxiety	Pearson correlation	0.270*	1	0.470**
	Sig. (two-tailed)	0.02		0.01
	*N*	980		980
Depression	Pearson correlation	0.346**	0.470**	1
	Sig. (two-tailed)	0.00	0.01	
	*N*	980	980	

**Correlation is significant at the 0.01 level (two-tailed). *Correlation is significant at the 0.05 level (two-tailed).

### Findings Related to Research Question 4

#### What Are the Levels of Anxiety and Depression Among Internet and Smartphone Users During COVID-19?

##### BAI and BDI

Mild mood disturbance was observed (BDI: 11–16: score) was classified as Mild mood disturbance band or range (Beck et al., 1996). The findings showed that the students with depression linked to happiness, pessimism, success/past failure, satisfaction, punishment feelings, disappointment, blame/self-criticalness, agitation, interest in people, worthiness, fatigue or tiredness, weight, and health. This is because they were linked to depression (Beck et al., 1996).

“Mild,” anxiety levels (8–15). This result was relatively different because some students reported that they had been infected with COVID-19, while others reported that they had not. Therefore, these results imply that further tests are needed to determine whether these symptoms appear to be mostly panic-related, autonomic, neurophysiologic-related, or subjective.

#### Insights of Students’ Mental Health Aspects

The BAI and BDI examinations were conducted on a random sample of 10 students to determine their anxiety and depression levels because of COVID-19 and understand how digital education and multidimensional perfectionism interacted to influence their mental health.

#### Insights Into Students’ Anxiety Levels

Overall scores for all the 21 anxiety symptoms were computed and it was found that three students had “minimal” levels of anxiety (0–7: score 3; score = 4; and score = 5), and five students had “mild” anxiety levels (8–15: score = 9; score = 9: score 10; score = 12; and score = 13). On the other hand, 1 student was discovered to have a “Moderate,” anxiety level (16–25: score = 23). Only one student had a severe anxiety level (26–63: score = 29). These results reported that some students having been infected with COVID-19, while others were free from COVID-19 infections. Therefore, these results imply that further tests are needed to determine whether these symptoms appear to be mostly panic-related, autonomic, neurophysiologic-related, or subjective.

#### Insights Into Students’ Depression Levels

Two cases of moderate depression were observed in students (BDI: 20–29: score 21; score 29). The remaining cases were classified as mild as they were within the 11–16 severity range (Beck et al., 1996). The findings showed that the students required special attention in dealing with depression linked to happiness, pessimism, success/past failure, satisfaction, punishment feelings, disappointment, blame/self-criticalness, agitation, interest in people, worthiness, fatigue or tiredness, weight, and health. This is because they were linked to depression (Beck et al., 1996).

#### Indirect or Mediating Effects

Indirect effects were computed with the aim of identifying the mediating effects of the digital environment and multidimensional perfectionism on students and non-students’ mental health. This was accomplished by examining the indirect effects of COVID-19 on students and non-students’ mental health.

#### COVID-19’s Indirect Effects on Students’ Mental Health

A significantly low and positive coefficient of.135 linking COVID-19, digital environment, and mental health was observed. This denotes that the digital environment has positively mediating effects on the relationship between COVID-19 and mental health among students. However, it was noted that multidimensional perfectionism has negatively mediating effects on the relationship between COVID-19 and mental health among non-students (β = –0.155; *p*-value = 0.000).

#### COVID-19’s Indirect Effects on Non-students’ Mental Health

According to Table 6 a low positive and Significant indirect effect of 0.310 was observed between COVID-19, digital environment, and mental health (*p*-value < 0.05). Therefore, this led to the conclusion that the digital environment has positive mediating effects on the relationship between COVID-19 and mental health among non-students. On the other hand, a relatively high and significant indirect effect of 0.545 was observed between COVID-19, multidimensional perfectionism, and mental health (*p*-value < 0.05). Hence, it was accepted that multidimensional perfectionism has positive mediating effects on the relationship between COVID-19 and mental health among non-students.

**TABLE 6:** COVID-19’s indirect effects on non-students and students’ mental health.

Non-student	Original sample	Standard deviation	T statistics	*P-values
COVID-19 - > Digital environment - > Mental health	0.310	0.039	7.987	0.000
COVID-19 - > Multidimensional perfectionism - > Mental health	0.545	0.147	3.708	0.000

**Student**	**Original** **sample**	**Standard** **deviation**	***T* statistics**	*P-values

COVID-19 - > Digital environment - > Mental health	0.135	0.061	2.199	0.028
COVID-19 - > Multidimensional perfectionism - > Mental health	–0.155	0.028	5.474	0.000

### Path Analysis

One of this study’ prime aim was to the COVID-19’s effects on digital environment and the presented findings show that COVID-19’s has significant positive effects on digital environment among students (β = 0.747; *p*-value = 0.000) and non-students (β = 0.432; *p*-value = 0.000). This implies that COVID-19 causes students to adopt online learning platforms, and non-students to adopt online work-from-home platforms.

Such is relatively true and aligns with several studies showing that COVID-19 has caused a significant adoption of internet-based applications by educational institutions (Salinas et al., 2017; Siron et al., 2020) and business organizations to avoid getting COVID-19 (He et al., 2021). Thus, hypothesis 1 (H1) was valid for both students and non-students.

Table 7’s Path analysis results also show that hypothesis 2 (H2) was valid for non-students as it adversely affected their mental health by –0.288. However, contrasting results were observed for students as COVID-19 could be seen as having a positive effect on students’ mental health by 0.378. This suggests that non-students could have been worried more about family obligations, increased burdens, and other related responsibilities, which were adversely affected by COVID-19 compared to students who might bear the same responsibilities and/or consequences (see Table 7).

**TABLE 7:** Path analysis.

Student non-student				
	Coefficient	*P*-value	Coefficient	*P*-value
COVID-19 - > Digital environment	0.747	0.000	0.432	0.000
COVID-19 - > Mental health	0.378	0.000	–0.288	0.033
COVID-19 - > Multidimensional perfectionism	0.285	0.000	0.857	0.000
Digital environment - > Mental health	0.181	0.027	0.717	0.000
Multidimensional perfectionism - > Mental health	–0.544	0.000	0.636	0.000

Table 7 shows that COVID-19 had positive effects on students and non-students’ multidimensional perfectionism by 0.285 and 0.857, respectively. Thus, hypothesis 3 (H3) was accepted and this implied that the prevalence of COVID-19 caused students and non-students to improve their socially prescribed, self-oriented, and other-oriented perfectionism (Hewitt and Flett, 1989). Such is vital in guarding against the spreading of COVID-19.

The students’ path diagram and hypothesis 4 (H4) was accepted and this implied that digital environment improvements help to improve students and non-students’ mental health by 0.181 and 0.717, respectively. This also shows that non-students mental health was greatly enhanced by digital environment improvements during COVID-19 compared to students.

**FIGURE 1:**
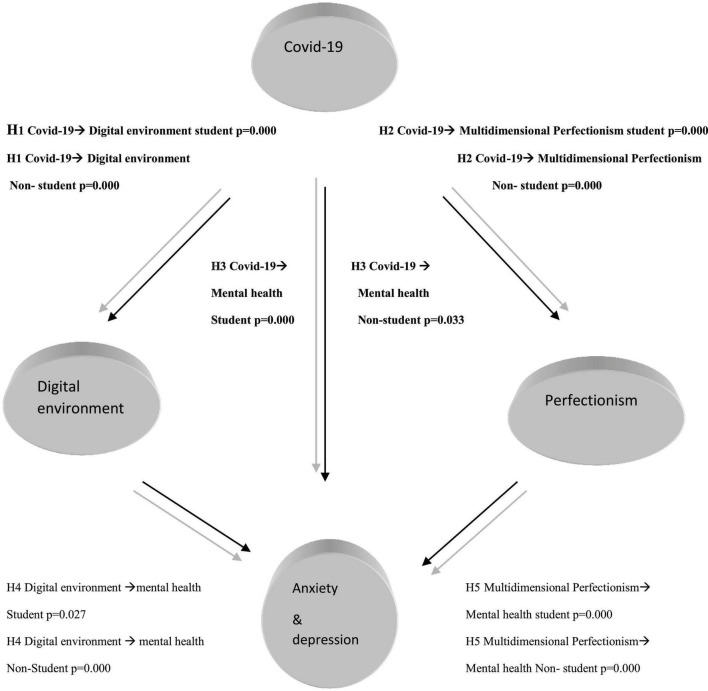
Graphical illustration of the Path analysis.

Thus, the adoption of digital platforms by individuals, educational institutions, and business organizations assisted in avoiding getting COVID-19 (Salinas et al., 2017; Siron et al., 2020; He et al., 2021). As a result, this deals away with the fear of having to get COVID-19, and, thus, improves their mental health. The students and non-students’ path analysis results can be summarized using Figure 1.

Improvements in multidimensional perfectionism had adverse effects on students’ mental health by –0.544 and positive effects on non-students’ mental health by 0.636. This positively indicates that the students’ socialization abilities were negatively affected and were prevented from getting access to classroom learning platforms and personal interactions with their teachers. Besides, getting used to online learning platforms requires adjustments, which most students were not used and, thus, adversely affected their mental health. The opposite is valid for non-students who used socially prescribed, self-oriented, and other-oriented multidimensional perfectionism aspects to their advantage and avoided getting COVID-19. This was vital for enhancing their mental health and, hence, hypothesis 5 (H5) was accepted for non-students and rejected for students’ scenario. The non-students’ path analysis results can be summarized using Figure 1.

#### Model Fit

In Table 8 no discrepancies were observed between the saturated and estimated models, as noted by the SRMR values, which were below 0.8. Both d_ULS and d_G were insignificant at 0.05. While the Chi-square values were significant and the NFI values were above 0.70, and this indicates that the model was fit for fulfilling this study’s intended purpose of examining the effectiveness of digital environment and perfectionism on psychological disorders (anxiety and depression) during COVID-19 among students and non-students.

**TABLE 8:** Model fit.

Student			Non-student	
	Saturated model	Estimated model	Saturated model	Estimated model
SRMR	0.059	0.059	0.109	0.109
d_ULS	0.888	0.891	0.786	0.776
d_G	0.310	0.309	0.420	4.183
Chi-Square	900.408*	898.477*	436.106*	432.563*
NFI	0.845	0.846	0.845	0.846

*Significant at 0.05 level.

## Self-Reported Results

### Findings Related to Qualitative Research Question 1

A thematic approach was applied to code and analyze the self-reported results using Nvivo software. The report result indicates the views of internet and smartphone users about the effectiveness of digital environment on anxiety and depression during COVID-19. In total, 45 students and 41 non-students indicated that they had good perspective about the role of the digital environment during COVID-19 in targeting anxiety and depression. On the contrary, three students and four non-students highlighted that they had bad attitudes, while two students and five non-students had mixed assumptions about the role of the digital environment during COVID-19 to target anxiety and depression. This suggested that both students and non-students largely anticipated that digital environment improvements would play a vital role in avoiding COVID-19 infections and dealing with problems posed by COVID-19. This is further supported by results of the interviews, in which both students and non-students were asked if they thought that digital environment would change their thoughts and emotions. All the students and non-students agreed, and, thus, this further reinforces that digital environment improvements are essential and are highly welcomed in Kurdistan.

### Findings Related to Qualitative Research Question 2

Secondly, the interview process sought to determine the views of internet and Smartphone users about the influence of perfectionism in the time of the coronavirus. Both students and non-students agreed that they determined perfectionism as the away of protecting themselves during the COVID-19 health crisis this highlights the importance of socially prescribed, self-oriented, and other-oriented perfectionism in curbing COVID-19 infections (Hewitt and Flett, 1989). Additionally, both sets of interviewees agreed that perfectionism has not led to any form of psychological disorders during COVID-19. This suggests that encouraging individuals to adopt that perfectionism is crucial in dealing with COVID-19 infections and related psychological effects.

### Findings Related to Qualitative Research Question 3

Another aim of the interviews was to establish the views of internet and Smartphone users about the digital environment during COVID-19. As such, it was noted that all the students and non-students regarded or evaluated the role of the digital environment during COVID-19 as positive. This aligns with the previous aim’s findings and is supported by related findings showing that digital environment improvements are vital and will go a long way in preventing both the spread and effects of COVID-19 (Szopiński, 2016; Siron et al., 2020; He et al., 2021). Additional information obtained revealed that the digital environment was regarded as being beneficial for avoiding COVID-19 infections (*S* = 41; *NS* = 39), convenience (*S* = 9; *NS* = 5), and innovative purposes (*NS* = 6). However, the findings showed that the digital environment was disadvantageous because of costs related to its adoption (*S* = 24; *NS* = 28), interruptions (*S* = 17; *NS* = 15), and the unproductiveness its causes (*S* = 9; *NS* = 7).

### Findings Related to Qualitative Research Question 4

Similarly, the goal of conducting the interviews was to understand how perfectionism influences anxiety and depression during COVID-19. No concrete evidence was found as to whether self-evaluation performed by perfectionists led to anxiety and depression. Some interviewees responded that self-evaluation performed by perfectionists leads to anxiety and depression (*S* = 22; *NS* = 21), some said that self-evaluation done by perfectionists does not lead to anxiety and depression (*S* = 20; *NS* = 23), while others were uncertain as to whether self-evaluation done by perfectionists leads to anxiety and depression (*S* = 8; *NS* = 6). Nevertheless, students and non-students suggested that there is a consensus of self-evaluation that is done by perfectionist’s leads to anxiety and depression. On the other hand, perfectionists show symptoms that have been existed among anxious and worried people in the pandemic (*S* = 44; *NS* = 48).

### Findings Related to Qualitative Research Question 5

Lastly, the goal was to further explore the views of internet and smartphone users about the relation between digital environment, perfectionism, and anxiety and depression during COVID-19. Both students and non-students responded by suggesting that there is a relationship between digital environments, perfectionism, and anxiety and depression during COVID-19. This supports the argument put forward by this study and justifies its academic and practical necessity and importance. This can further be supported by the established views regarding the interviewees’ assessment about the connection between digital environments, perfectionism, and anxiety and depression during COVID-19, which were strong (*S* = 46; *NS* = 46), weak (*S* = 2; *NS* = 3), and moderate (*S* = 2; *NS* = 1).

Similarly, the goal of conducting the interviews was to understand how perfectionism influences anxiety and depression during COVID-19. No concrete evidence was found as to whether self-evaluation performed by perfectionists led to anxiety and depression. Some interviewees responded that self-evaluation performed by perfectionists leads to anxiety and depression (*S* = 22; *NS* = 21), some said that self-evaluation done by perfectionists does not lead to anxiety and depression (*S* = 20; *NS* = 23), while others were uncertain as to whether self-evaluation done by perfectionists leads to anxiety and depression (*S* = 8; *NS* = 6). Nevertheless, students and non-students suggests that there is a general consensus of self-evaluation that is done by perfectionist’s leads to anxiety and depression. On the other hand, perfectionists symptoms having been showed considerably among the people that anxious and worried concerning the pandemic, as well as the situation they find themselves in (*S* = 44; *NS* = 48) participants and against related contrary suggestions (*S* = 6; *NS* = 2) participants.

## Summary of Findings

In this section of the research, a summary of all the analysis was presented below.

Findings show that the adoption of digital education in the Kurdistan region of Iraq was primarily influenced by economic development, technological innovation, and structural factors like COVID-19. Also, the findings in this study, presented in the analysis above, show that the students’ multidimensional perfectionism was not influenced by their location. While, considerable differences between students and non-students were observed, as location significantly influenced multidimensional perfectionism among non-students. On the other side, the independent *t*-tests revealed that there were insignificant differences in depression levels among students. However, the students were significantly anxious about COVID-19 as the results show that the students’ anxiety levels measured by the BAI were significantly different.

In other words, the location effects of COVID-19 on non-student’s anxiety levels were insignificant. Because the digital environment helps students to acquire more information about COVID-19, anxiety, or depression. This causes them to adopt effective measures to reduce them and subsequently improves their mental health. The study suggests that digital environment improvements were being used for non-productive activities or purposes that adversely affected non-students’ mental health. This suggests that multidimensional perfectionism aspects, especially self-orientation of an individual’s this issue affect their well-being and happiness.

The findings showed that the students with depression linked happiness, pessimism, success/past failure, satisfaction, punishment feelings, disappointment, blame/self-criticalness, agitation, interest in people, worthiness, fatigue or tiredness, weight, and health and this result was relatively different because some students reported that they had been infected with COVID-19, while others reported that they had not. Therefore, these results imply that further tests are needed to determine whether these symptoms appeared to be mostly panic-related, autonomic, neurophysiologic-related, or subjective, both the students and non-students responded by suggesting that there is a relationship between digital environments, perfectionism, and anxiety and depression during COVID-19.

## Discussion

The goal of this study is to investigate the effects of perfectionism on psychological disorders (anxiety and despondency), as well as the effectiveness of the digital environment during the coronavirus COVID-19. Overall, the findings show that in this population, high levels of perfectionism are linked to high scores on a broad measure of psychological distress markers. Perfectionism was found to have a significant positive relationship with (a) sadness, (b) anxiety, and (c) stress in studies. This link existed regardless of where you were. Furthermore, in this sample, one perfectionism facet, doubts about actions, was consistently and specifically linked to psychological suffering and negative affective states.

In a similar vein, the study discovered both students and non-students in Iraq’s Kurdistan region suffer from moderate to high degrees of depression, anxiety, and stress, which is greater than normal levels. The figures indicate a risk of psychological disorders and suggest that academic and psychosocial performance will deteriorate throughout the quarantine time at home. One cause could be the unexpected onslaught of academic online assignments and sessions, compounded with social anxiety of death and the COVID-19 outbreak. Another factor could be related to students’ anxieties about their grades and graduation-related issues, as suggested by Sahu (2020), who has highlighted fear issues among college students. In a normal context, academic work is a cause of concern (Harper Shehadeh et al., 2020); however, COVID-19’s global apprehension and anxiety may have exacerbated the psychological status of both students and non-students. Students who do not have access to the necessary information technology to complete their online academic obligations are more likely to experience depression, anxiety, and stress. This has also been demonstrated by the negative connections of sadness, tension, and anxiety with students’ income levels, implying that their financial status has an impact on their psychological well-being.

According to lifecycle studies, non-students are more prone to acquire attachment and worry associated to the fear that family members would contract the disease, as well as opposition-challenging behaviors. Younger students have higher levels of depression, which has been linked to deprivation of liberty and school closures, whereas older students have depression linked to decreased activity level, sleep quality, well-being, and cognitive functioning, as well as widowers and separated people having a higher risk of developing emotional disorders during the COVID-19 pandemic.

Another probable explanation for the lower mental health during COVID-19 is COVID-19 information overload, which has been marked by inconsistent information from various international and local agencies, experts and scientists from various backgrounds, and the media. COVID-19 information is frequently updated and obtained *via* social media platforms such as Twitter and Facebook. People, on the other hand, have been overwhelmed by the amount of COVID-19 information they have received.

According to a recent study conducted in mainland China, more social media exposure increased the risk of feeling anxiety. In fact, past research has demonstrated that media exposure to mass trauma can result in post-traumatic stress disorder. Furthermore, during a Middle East respiratory syndrome coronavirus outbreak in South Korea, a recent study indicated that social media exposure was positively connected to creating risk perceptions (Choi et al., 2017). It is a two-edged sword when it comes to social networking. They have the ability to quickly distribute critical information, so that individuals can take proper public precautions to protect themselves. However, rumors, misinformation, and fear can also readily spread through social media, further heightening fear and anxiety.

Finally, this study found that university students in Iraq’s Kurdistan region experienced high levels of anxiety during the COVID-19 pandemic. The student’s anxiety levels were significantly reduced by quarantine and switching to a distance learning technique. As a result, legislators should examine student mental health in order to develop a support programmer to help students improve their mental health. Students must undergo screening before enrollment in order to detect risk factors. We discovered that sleep and eating disorders, anxiety over academic results, and feelings of loneliness are all key factors that mental health nurses and counselors at academic institutions and counseling departments must address and care for. Faculty and administrators must also provide proper psychological support and avoid overburdening students during quarantine periods.

## Conclusion

The study revealed interesting aspects about the effectiveness of digital environment and perfectionism on anxiety and depression in times of the COVID-19 pandemic crisis. Death rate has increased very high during the COVID-19 pandemic situation, leading the governments with no choice than imposing some measures to reduce the rapid spread of the COVID-19 disease. These measures may include lockdown of major activities in every city, compulsory quarantine of travelers, social distancing in markets and shopping malls, etc. These also accumulated to the overall problems of anxiety and depression of both students and non-students in Northern Iraq. The digital environment affects both the students and non-students in both positive and negative ways, because it was very effective in providing platform for virtual communication and enhancing social distance; while on the negative part, it also lead to anxiety, depression, and psychological issues during the beginning of the pandemic period. Relevant literatures were revised and the results were also reviewed along with survey using questionnaire in this study. The review of previous studies found that smartphones has both importance and problems attached to it. About 92% of adolescent students around 14–17 years engaged in social media activities; while 15–17 years range of students uses smartphones and 68% of students aged 13–14 also uses smartphones. This is linked both positively and more toward negative consequences looking at the anxiety and depression factors smartphones can cause. The COVID-19 pandemic period brought about the growth of the digital environment, particularly the internet and smartphones, realizing several psychological effects attached to it. Result show that participants internet use of nearly 9 h per day is a long time, according to the internet use standard, so it is important to publish awareness about internet risk factors for health and psychological, with that, it is important result to ministry of health and higher education to use this data to build a strong program to overcome internet addiction. Another finding indicate the level of depression and anxiety among participant with mild level, thus, giving a good reason for other organization, foundation, and government to work toward this issue among population by special program for parents, educator and families. Also, do more relate research about this problem? This study concluded by revealing that digital environment and perfectionism have affected anxiety and depression during COVID-19. However, the study also showed that participants had mild levels of depression and anxiety. As pointed out by other studies, these two variables could create an environment which unsupported individualism and discourages the sharing of knowledge amongst students and non-students. However, the studies show that there is a relationship between digital environments, perfectionism, and anxiety and depression during COVID-19 amongst students and non-students in northern Iraq.

### Clinical Implications

Perfectionism was strongly connected with the Digital environment, thus, this personality characteristic should be addressed in the creation of tailored treatments to avoid overuse of the internet and Smartphone users, according to the findings of the current study. Perfectionism is said to be formed during the socialization process and is regarded to be a stable personality trait in adulthood (Flett et al., 2011). Furthermore, the significance of despair and anxiety in mediating the relationship between perfectionism and excessive internet and Smartphone usage led to the conclusion that mental health education for college students is crucial in preventing excessive internet and Smartphone use. Additionally, therapy interventions could be developed to improve students’ ability to cope with negative emotions, thus, preventing the self-medication process. Stress Temperament Coaching is an intervention model that might be customized and utilized in (Munakata, 2007). It has been shown to be helpful in reducing psychological discomfort by encouraging active coping.

According to the internet use standard, participants use the internet for nearly 9 h per day, which is a significant amount of time. As a result, it is critical to raise awareness about internet risk factors for health and psychological issues. Additionally, it is critical for the ministry of health and higher education to use this data to develop a strong program to combat internet addiction.

Another finding indicates that participants with mild depression and anxiety, which provides a good reason for other organizations, foundations, and the government to work on this issue among the population during COVID-19 to prevent or minimize psychological problems through special programs for parents, educators, and families.

### Limitation of the Study

One of the drawbacks is that this study did not include cross-sectional design, which means that there was no reported information about pervious or current treatments (both medications (pharmacological and psychotherapeutic session), while standardized instruments have been adapted, we do not have psychometrics and validation’s specific information. Lack of ample information about methods of treatment (like psychotherapeutic, psychopharmacological) that are necessary for those who participated and did not. Psychotherapy is a critical component of the COVID-19 crisis response. As psychotherapists, we are especially able to discuss the psychological consequences of social isolation, job loss, fear of infection, and grieving with our patients. It is both our privilege and job to assist patients who are experiencing huge life changes as a result of the pandemic, assisting them in coping with the chaos that has been foisted upon us all. They may be seen, heard, and remembered when they are with us (Swartz, 2020). On the other hand, in the pharmacologic treatment of panic disorder, selective serotonin reuptake inhibitors (SSRIs), and benzodiazepines (BZs) are beneficial to panic disorder (PD) (Quagliato et al., 2019). The research is restricted only to smartphone and internet users and, thus, during COVID-19, the result cannot be extended across all the population of the digital environment. The research mentions and reflects on fear and depression two psychological disorders among all psychological disorder that can be experienced by individuals as a side effect of COVID-19. Participants may also hesitate to discuss their psychological state, which may impact the study’s outcomes and findings.

The effect of the digital environment and perfectionism has been analyzed based on anxiety and depression levels during COVID-19 pandemic. This means that the aim of the study has been attained and recommendation were stated here.

The following recommendation can be made on the study findings:

Most importantly, an urgent investigation is required to investigate technical applications throughout the post-pandemic instead of throughout the early stages of the pandemic.

Future research should study dissimilar impacts of technology application, particularly dissimilar undesirable impacts, such as misrepresentation and deception. This is because it is known that incorrect news and information are spread quickly and sometimes have adverse effects on people’s psychology and physiology, so it is important to conduct more studies on people’s belief in social media news.

## Data Availability Statement

The original contributions presented in the study are included in the article/supplementary material, further inquiries can be directed to the corresponding author/s.

## Ethics Statement

The studies involving human participants were reviewed and approved by Near East University Ethical Committee Board. The patients/participants provided their written informed consent to participate in this study.

## Author Contributions

Both authors listed have made a substantial, direct, and intellectual contribution to the work, and approved it for publication.

## Conflict of Interest

The authors declare that the research was conducted in the absence of any commercial or financial relationships that could be construed as a potential conflict of interest.

## Publisher’s Note

All claims expressed in this article are solely those of the authors and do not necessarily represent those of their affiliated organizations, or those of the publisher, the editors and the reviewers. Any product that may be evaluated in this article, or claim that may be made by its manufacturer, is not guaranteed or endorsed by the publisher.
